# Variation in the fitted filtration efficiency of disposable face masks by sex

**DOI:** 10.1038/s41370-024-00697-4

**Published:** 2024-07-02

**Authors:** Edward R. Pennington, Jacob S. Griffin, E. Melissa McInroe, William Steinhardt, Hao Chen, James M. Samet, Steven E. Prince

**Affiliations:** 1 Public Health and Integrated Toxicology Division, Center for Public Health and Environmental Assessment, Office of Research and Development, U.S. Environmental Protection Agency, Research Triangle Park, NC, USA.; 2 Oak Ridge Institute for Science Education, Oak Ridge, TN, USA.; 3 Department of Occupational and Environmental Health, School of Public Health, Guangxi Medical University, Guangxi 530021, China.; 4 Public Health and Environmental Systems Division, Center for Public Health and Environmental Assessment, Office of Research and Development, U.S. Environmental Protection Agency, Research Triangle Park, NC, USA.

**Keywords:** Face Masks, COVID-19, Air Pollution, Wildfire Smoke, Public Health, Mask Modification

## Abstract

**BACKGROUND & OBJECTIVE:**

Disposable face masks are a primary protective measure against the adverse health effects of exposure to infectious and toxic aerosols such as airborne viruses and particulate air pollutants. While the fit of high efficiency respirators is regulated in occupational settings, relatively little is known about the fitted filtration efficiencies of ear loop style face masks worn by the public.

**METHODS:**

We measured the variation in fitted filtration efficiency (FFE) of four commonly worn disposable face masks, in a cohort of healthy adult participants (*N* = 100, 50% female, 50% male, average age = 32.3 ± 9.2 years, average BMI = 25.5 ± 3.4) using the U.S. Occupational Safety and Health Administration Quantitative Fit Test, for an N95 (respirator), KN95, surgical, and KF94 masks. The latter three ear loop style masks were additionally tested in a clip-modified condition, tightened using a plastic clip to centrally fasten loops in the back of the head.

**RESULTS:**

The findings show that sex is a major determinant of the FFE of KN95, surgical, and KF94 masks. On average, males had an 11% higher FFE relative to females, at baseline testing. We show that a simple modification using an ear loop clip, results in improvements in the average FFE for females but provides comparatively minor changes for males. On average, females had a 20% increased FFE when a clip was worn behind the head, relative to a 6% increase for males.

**IMPACT:**

- The efficacy of a disposable face mask as protection against air contaminants depends on the efficiency of the mask materials and how well it fits the wearer. We report that the sex of the wearer is a major determinant of the baseline fitted filtration efficiency (FFE) of commonly available ear loop style face masks. In addition, we show that a simple fit modifier, an ear loop clip fastened behind the head, substantially improves baseline FFE for females but produces only minor changes for males. These findings have significant public health implications for the use of face masks as a protective intervention against inhalational exposure to airborne contaminants.

## Graphical Abstract

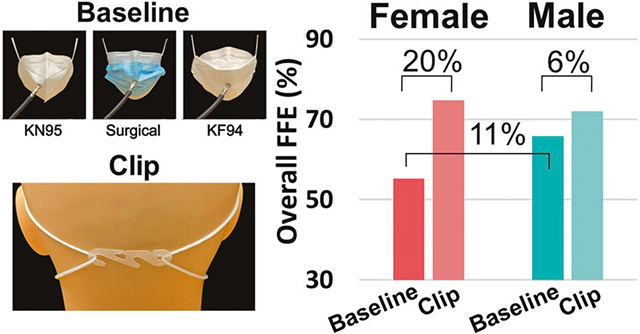

## INTRODUCTION

Disposable face masks are a primary intervention used to protect against aerosolized air contaminants, including respiratory viruses and wildfire smoke [1–3]. The use of face masks has been mainly limited to public health emergencies and occupational settings where inhalational hazards pose health risks [4–6]. The COVID-19 pandemic coincided with an unprecedented increase in the use of disposable face masks by the public. With the increased frequency and severity of wildland fires across the world, there has been even greater public awareness and adoption of disposable face masks as protection against wildfire smoke particulates. However, face masks that are commonly available to the public offer varying levels of protection against air pollutants [7, 8], due to intrinsic characteristics such as design, materials, and fabrication. In addition, multiple factors specific to the wearer contribute to the fitted filtration efficiency (FFE) of disposable face masks including, but not limited to training, facial hair, and facial variation [9–14]. In the U.S., N95 respirators remain a benchmark for disposable respiratory protection, offering filtration efficiencies of 95% and greater (NIOSH-42CFR84) [15]. In contrast, there is limited data, on the effectiveness and efficacy of the more commonly worn disposable face masks such as KN95, surgical, and KF94 types.

In the U.S., access to mask fit testing is limited to occupational settings for workers whose positions require respiratory protection. These respirators are certified by the National Institute of Occupational Safety and Health (NIOSH) and are fitted using testing protocols sanctioned by the Occupational Safety and Health Administration (OSHA). Thus, face mask materials can be certified to specific levels of protection (e.g., N95, P100), but may differ in fitted performance as worn by individuals. The OSHA quantitative fit test is used to determine whether a specific respirator model can achieve performance standards necessary to protect an individual worker from workplace exposure. Due to lack of industry regulations and certifications for ear loop style disposable face masks, the public is left to rely on perceived effectiveness when selecting a mask. As such, adoption of disposable face masks by the public has been largely driven by individual factors, such as comfort, convenience, and availability. During the COVID-19 pandemic KN95 and surgical masks, together, comprised the most commonly worn disposable face masks by the public, with KF94 masks and N95 respirators representing less common options in the United States [16]. Lacking public access to standardized mask fit testing also means that data on the range of protection provided by the most common face masks is largely nonexistent.

This data gap has left the public uncertain about the level of protection provided by different masks and whether they would benefit from measures to improve their fit. During the COVID-19 pandemic, a variety of modifications or “hacks” were developed to improve the fitted performance of disposable face masks [8, 9, 12, 17]. Many of these modifications aim to improve mask fit by reducing gaps between the mask and face. The primary goal of this study was to investigate the variation in FFE of disposable ear loop style respiratory masks commonly worn by the public (i.e., KN95, surgical mask, and KF94), relative to the benchmark represented by NIOSH certified respirators (N95). In addition, we aimed to determine whether a simple modification, using an ear loop clip fastened behind the head, improves the FFE of these masks. We report the combined and sex-stratified FFE for all masks tested at baseline, as well as the magnitude of FFE improvement in the clip-modified condition, showing striking differences between males and females.

## METHODS

### Study participants

This study was reviewed and approved by the University of North Carolina at Chapel Hill Institutional Review Board and the U.S. Environmental Protection Agency Human Subjects Safety Review Officer. Healthy participants (*N* = 100, 50% female, 50% male, average age = 32.3 ± 9.2 years, average body mass index (BMI) = 25.5 ± 3.4 – see Table 1 for participant demographics) were recruited to participate in this study. Participants self-reported their age, gender, sex assigned at birth, and race/ethnicity. Reported gender matched assigned sex at birth for all participants, thus males and females were classified using their biological sex. Participant height, weight, and blood pressure were obtained by certified registered nurses. Participants were excluded if their BMI was below 19.0 or above 33.4, or if they had a history of cardiovascular disease, chronic respiratory disease, cancer, uncontrolled hypertension ( ≥140 mmHg systolic, ≥90 diastolic mmHg), or diabetes. Thirteen participants, nearly evenly split between BMI and hypertension, fell beyond the exclusion criteria. All participants were required to be clean shaven to reduce potential variability associated with facial hair [13].

### Testing procedures

Following medical evaluation and anthropometric data collection, participants performed a modified version of the Occupational Safety and Health Administration (OSHA) Quantitative Fit Testing Protocol (Modified Ambient Aerosol CNC Quantitative Fit Testing Protocol for Filtering Facepiece Table A–2—RESPIRATORS) [15] in an atmosphere supplemented with aerosolized sodium chloride particles (mean diameter ~50 nm), produced by a TSI 8026 Particle Generator (TSI, Shoreview, MN). A pair of condensation particle counters (CPC – TSI model 3775) continuously monitored particle counts per cubic centimeter in the ambient chamber air and breathing space behind-the-mask with 1 second resolution, as previously described [9]. Each test lasted approximately 2–3 minutes and involved the following disposable face masks: a tri-fold 3M N95 Aura 9205+ (3M, St. Paul, MN, USA), a KN95 (Zhongshan Saifute Labor Protective Articles Co., Guangdong, China), a 3-ply surgical mask (Hannah Linen, Portland, OR, USA), and a size large KF94 (Dr. Puri, KM Corporation, Gyeonggi-do, Republic of Korea). The ear loop style masks were chosen to be broadly representative of the most common mask types worn in the U.S. by the general public during the COVID-19 pandemic [16]. Mask manufacturer was chosen based on availability and ease of higher-volume purchase from allowed vendors. Each mask was fitted with an aluminium port and connected to the CPC using a stainless steel Luer lock connector and conductive tubing. In Experiment 1, face masks were tested to investigate the primary goal of quantifying variation in baseline FFE. A study team member assisted in correcting any donning errors to reduce any visible gaps prior to testing [9]. In Experiment 2, the clip-modified condition entailed tightening the ear loops by fastening a clip behind the head of the wearer centered between the ears and secured at the nearest linkage points relative to its edges (clip dimensions: 5.3 cm long × 1.5 cm wide; see graphical abstract).

### Statistical analysis

Fitted filtration efficiency (FFE), calculated as (100 × [1 − particle number in breathing space behind mask / particle number in ambient chamber air]), was the primary outcome variable for each participant and each mask testing session. Experiment 1 used the FFE results for each mask at baseline to assess variation relative to the overall average FFE. Experiment 2 used only ear loop mask values at baseline and under clip-modified conditions to assess FFE improvement/change (delta).

For experiment 1, we performed a mixed effects linear regression to analyze the overall FFE for each mask relative to the overall average FFE across all masks. To account for individual differences in FFE, participant was included as a random effect in this model. We first used a base model to assess any significant difference between baseline FFE for each mask. We then improved the model by using mask type and sex, with an interaction term, as fixed effects. The equation to describe the relationship between overall FFE $\left(y_{i}\right)$ and the fixed effects is:

$$y_{i}=\beta_{0}+\beta_{1}x_{i1}+\beta_{2}x_{i2}+\beta_{3}x_{i3}+\beta_{4}x_{i4}+\beta_{5}x_{i5}+\beta_{15}x_{i1}x_{i5}+\beta_{25}x_{i2}x_{i5}\;\;+\beta_{35}x_{i3}x_{i5}+\beta_{45}x_{i4}x_{i5}+\epsilon_{i}$$

where $\beta_{0},\;\beta_{1},\;\beta_{2},\;\beta_{3},\;\beta_{4},\;\beta_{5},\;\beta_{15},\;\beta_{25},\;\beta_{35}$, and $\beta_{45}$ are regression coefficients and $\epsilon_{i}$ is the independent normal error term. $X_{1},\;x_{2},\;x_{3}$, and $x_{4}$ are the variables for N95, KN95, surgical, and KF94, respectively. The mask variable equals 1 when that mask is being tested or otherwise is 0. $X_{5}$ is sex which equals −1 for male and 1 for female. An interaction term between mask and sex is also included in the model. Post-hoc Tukey tests were performed to analyze pairwise comparisons between males and females for each mask. We then conducted sensitivity analysis by adding the variables age, BMI, and race/ethnicity to the mixed effects model.

Experiment 2 analyses followed a similar process with a mixed effects linear regression used to model the effect of modifying the ear loop masks with a clip. Participant was again included as a random effect while mask type, sex, and clip modification were fixed effects. The equation to describe the relationship between overall FFE $\left(y_{i}\right)$ and the fixed effects is as follows:

$$y_{i}=\beta_{0}+\beta_{1}x_{i1}+\beta_{2}x_{i2}+\beta_{3}x_{i3}+\beta_{4}x_{i4}+\beta_{5}x_{i5}+\beta_{45}x_{i4}x_{i5}+\epsilon_{i}$$

where $\beta_{0},\;\beta_{1},\;\beta_{2},\;\beta_{3},\;\beta_{4},\;\beta_{5}$, and $\beta_{45}$ are regression coefficients and $\epsilon_{i}$ is the independent normal error term. $X_{1},\;x_{2}$, and $x_{3}$, are the variables for KN95, surgical, and KF94 respectively. The mask variable equals 1 when that mask is being tested or otherwise is 0. $X_{4}$ is the variable for clip modification, where 1 is unmodified and −1 is modified. $X_{5}$ is the variable for sex which equals −1 for male and 1 for female. An interaction term between clip modification and sex is also included in the model. Sensitivity analysis was conducted as described for Experiment 1. Pairwise t-tests were also used to assess the differences of the change in FFE with the clip modification between males and females for each mask. To further explore the impact of the clip on modified FFE, participants were divided into baseline FFE quartiles for each mask. We then used mixed effects linear regression to estimate the difference between clip-modified and baseline FFE (delta = clip-modified − baseline). The equation to describe the relationship between delta $\left(y_{i}\right)$ and the fixed effects is as follows:

$$y_{i}=\beta_{0}+\beta_{1}x_{i1}+\beta_{2}x_{i2}+\beta_{3}x_{i3}+\beta_{4}x_{i4}+\epsilon_{i}$$

where $\beta_{1},\;\beta_{2},\;\beta_{3}$, and $\beta_{4}$ are regression coefficients and $\epsilon_{i}$ is the independent normal error term. $X_{1}$ is the baseline FFE for the participant while $x_{2},\;x_{3},\;x_{4}$, are the variables for quartile 2, 3 and 4, respectively. The quartile variables equal 1 if the participant is classified in that quartile for baseline FFE and 0 otherwise.

All analyses were conducted with R software (R Core Team (2021)), using the nlme (Linear and Nonlinear Mixed Effects Models) and emmeans (Estimated Marginal Means) packages. Overall FFE measures are given as the mean ± standard deviation. Linear regression results are presented as the effect estimate and associated 95% confidence interval. The best fit models were determined using Bayesian Information Criterion (BIC) values. *P* values < 0.05 were considered statistically significant for all models. Complete results for the described models are available in the Supplemental material.

## RESULTS

### Experiment 1: Fitted filtration efficiency (FFE) of commonly worn disposable face masks

**Fig. 1:**
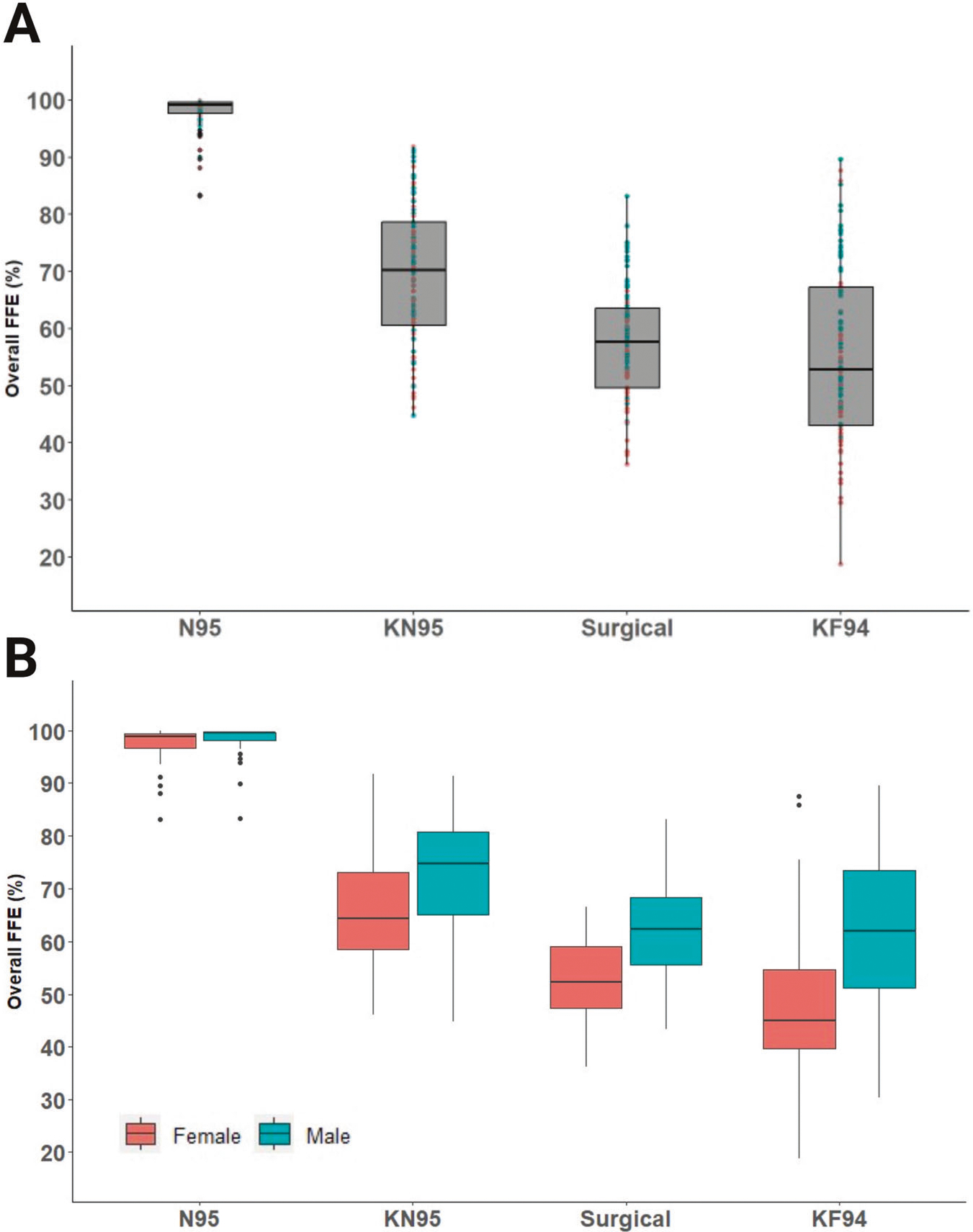
The fitted filtration efficiency (FFE) of commonly available face masks varies with sex. The overall FFE for the N95, KN95, surgical, and KF94 masks is shown in box and whisker (interquartile) plots for all study participants (*n* = 100; 50 females, 50 males) having performed the modified OSHA Quantitative Fit Testing Protocol (**A**), FFE stratified by sex (**B**). The overall FFE for each face mask was determined by the following expression: [1 − (mask count/ambient count)] × 100; which is shown as Overall FFE (%) plotted against the face mask type tested. Diamonds above or below the whiskers are flagged as statistical outliers.

Demographic characteristics and BMI (Kg/m^2^) of the study participants are listed in Table 1. The overall FFE values for all participants are illustrated in Fig. 1A and are shown separately for female and male participants in Fig. 1B. As expected, the N95 respirator provided the highest FFE and lowest standard deviation (97.8% ± 3.2). There was a significant difference in mean baseline FFE values of all masks and the overall mean FFE (*p* < 0.0001) except between KN95 (*p* = 0.43) (Table 2). The equation for the fixed effects is shown below. The random effect has a standard deviation of 5.5 for the intercept, corresponding to the variation between participants. (See supplemental material for full output from the model along with 95% confidence intervals).


estimatedFFE=70.0+27.9(N95)-0.58(KN95)-12.5(surgical)-14.8(KF94)-4.2(Sex)+3.8(N95*Sex)+0.505(KN95*Sex)-0.85(surgical*Sex)-3.422(KF94*Sex)

Age and BMI were significant variables in a follow-up sensitivity analysis while race/ethnicity was not. Inclusion of these variables did not confound the interpretation of the primary mixed effects model, which had the lowest BIC.

### Sex differences in disposable face mask baseline performance

Overall, the mean baseline FFE (Table 2; Fig. 1B) for all face masks tested was higher for males than for females. In the model, sex is a significant predictor for the response (*p* < 0.001). The following estimated mean FFE difference between females and males were significant −7.4% (95% CI −13.6, −1.1) for KN95, −10.1% (−16.3, −3.8) for surgical, and −15.2% (−21.5, −9.0) for KF94 masks. However, the difference between the mean FFE for females and males, −0.8% (−7.1, 5.4), for N95 was not significant.

### Experiment 2: The effect of a clip as a modifier of fit

**Fig. 2:**
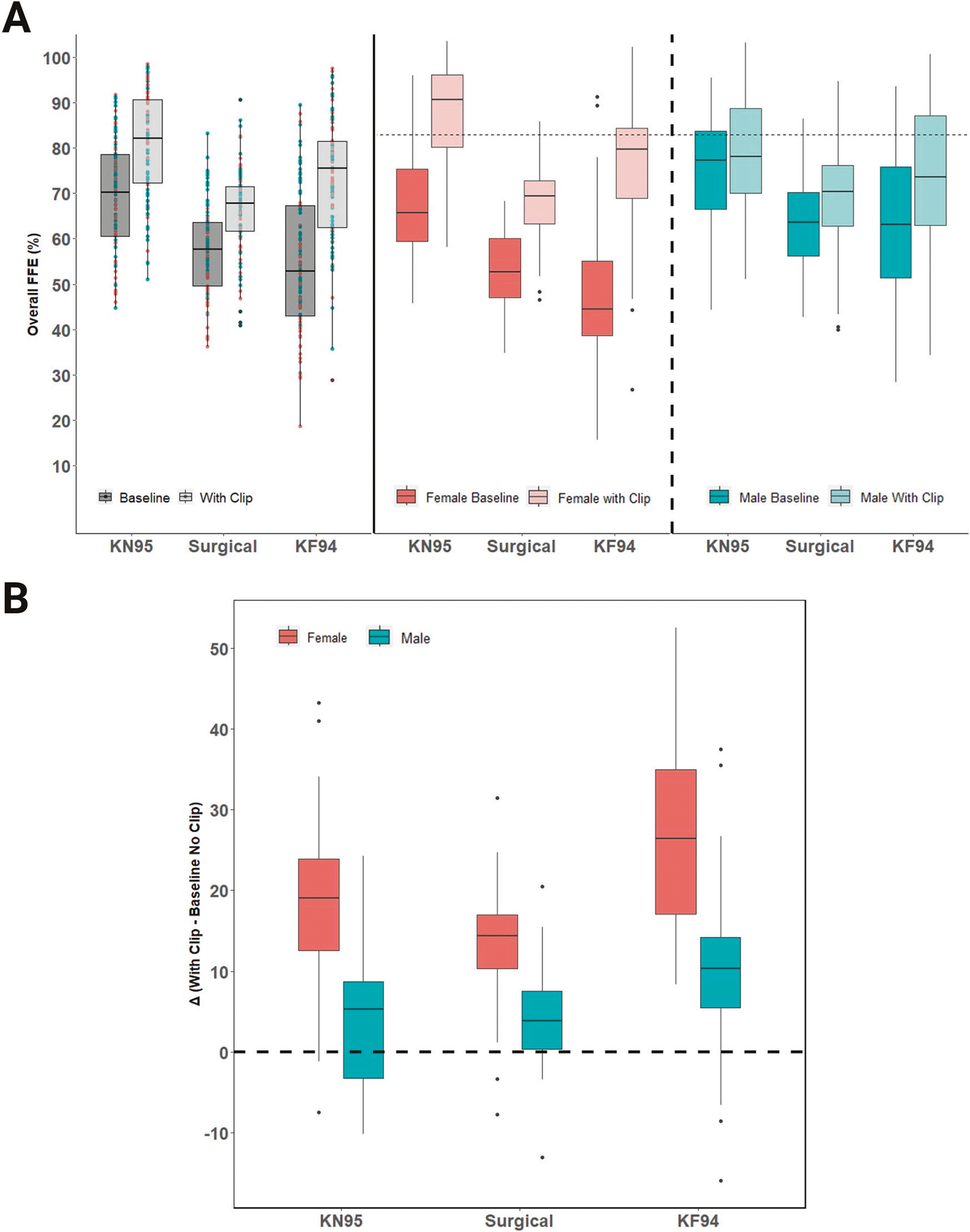
The fitted filtration efficiency (FFE) of ear loop masks changes with clip-modification. Box and whisker (interquartile) plots from 100 participants demonstrating improvement from baseline in the efficiency (FFE) of KN95, surgical, and KF94 masks following modification with an ear loop clip (**A**; left panel). Box and whisker plots data stratified by sex with a dashed line at 80% for reference (E.U. FFP1 material standard) (**A**; middle panel (female) and right panel (male)). Summarized change in FFE performance (delta) by mask and sex (**B**). Diamonds above or below the whiskers are flagged as statistical outliers.

The clip-modified condition resulted in a significant increase in average FFE (*p* < 0.0001), with an estimated mean difference for modified relative to baseline FFE of 6.4% (95% CI 5.7, 7.1). Figure 2A (left panel) shows the overall FFE for each mask at baseline and with the clip. Average and median FFE for each clip-modified mask and the difference (delta) compared to baseline are shown in Table 3. As with the unmodified masks, the modified KN95 (80.8% ± 12.0) had the highest average performance and an average delta of 11.4%. The surgical mask showed the least improvement with modified performance of 66.4% ± 9.4 for an overall average delta of 8.9%. The KF94, however, exhibited the largest numerical change when using the clip, with FFE rising to an average of 73.1% ± 13.9 for an overall average improvement (delta) of 17.9% across participants. Inclusion of additional variables in a sensitivity analysis did not confound the interpretation of the primary mixed effects model. The equation for fixed effects is shown below. The random effect has a standard deviation of 7.9 for the intercept corresponding to variation between participants.


estimatedFFE=67.0+8.0(KN95)-5.1(Surgical)-2.9(KF94)-6.4(Clip)-2.0(Sex)-3.4(Clip*Sex)

**Fig. 3:**
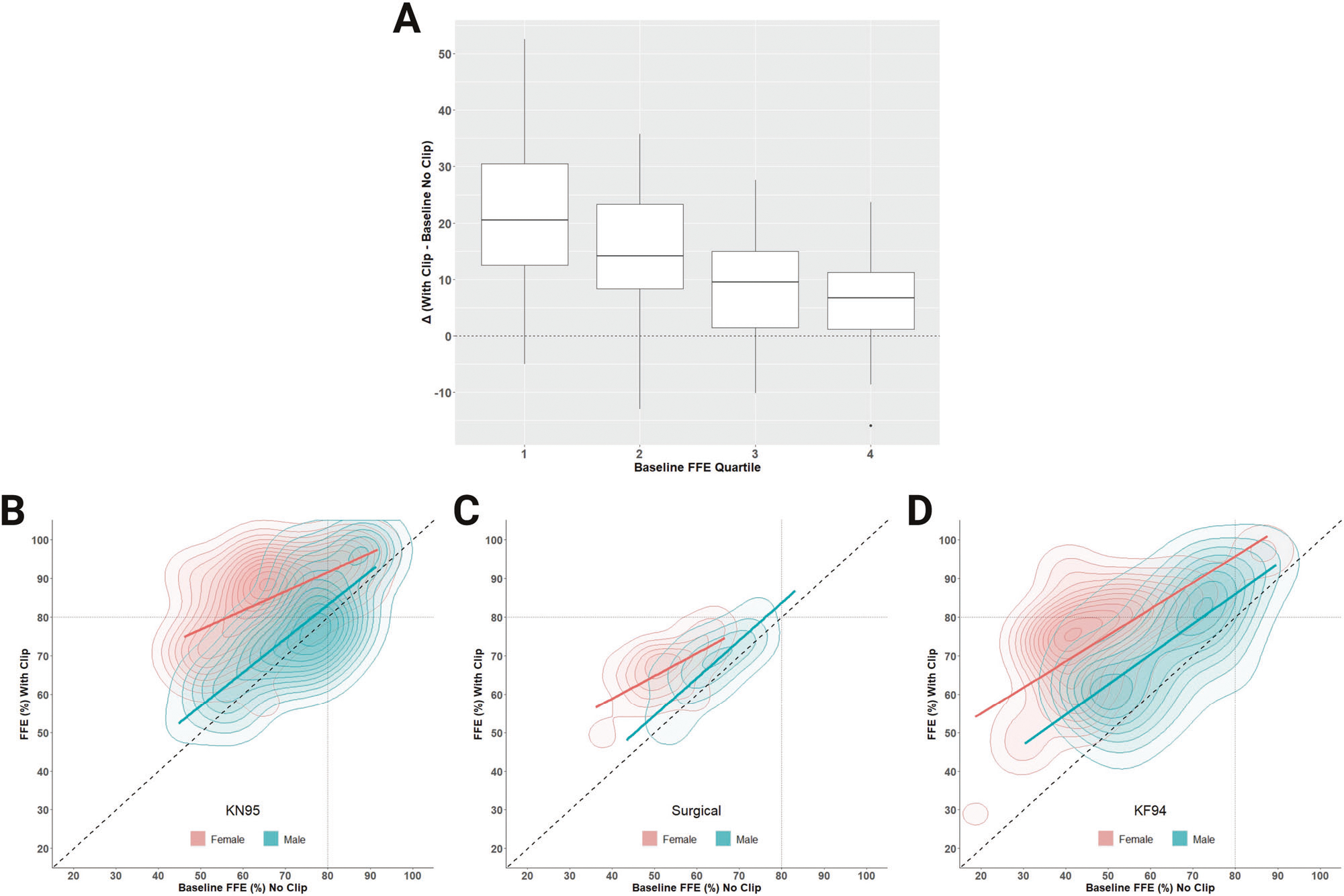
The change in fitted filtration efficiency (FFE) after clip-modification varies by baseline performance. Change with clip minus baseline (delta) across all ear loop masks, stratified by the baseline FFE quartile (*1*^*st*^ = <25%; *2*^*nd*^ = >25 to <50%; *3*^*rd*^ = >50 to <75%; and *4*^*th*^ = >75% segments of the distribution) (**A**). Cloud plots with FFE at baseline on the x-axis and clip-modified FFE on the y-axis (**B, C, D**). The diagonal line indicates the null hypothesis (no change) while contours above and below reflect FFE improvement and reductions, respectively. The 80% dashed lines are shown for reference (E.U. FFP1 material standard). Diamonds above or below the whiskers are flagged as statistical outliers.

When participant quartiles of baseline FFE (see Table 2) were considered, a significant effect was found for clip modification as a function of quartile. Figure 3A shows the average delta (across all ear loop masks) by quartile of baseline performance. The estimated magnitude of improvement was significantly greater (all *p* < 0.05) for the first quartile of baseline performance relative to the 2^nd^, 3^rd^, and 4^th^ quartiles. The equations for fixed effects below show negative coefficients for quartiles 2, 3, and 4 indicating a lower estimated mean delta. The random effect has a standard deviation of 5.8 for the intercept corresponding to variation between participants.


estimateddelta=31.58-0.24(FFE)-3.38(Quartile2)-6.40(Quartile3)-6.88(Quartile4)

### Sex Differences in clip-modified disposable face mask performance

In Fig. 2A (middle and right panels), the effect of an ear loop clip behind the mask on FFE is shown for females and males, respectively. The modification improved the fit and overall FFE for both females and males; however, the greatest improvement over baseline was observed for female participants (up to 13.7–26.1% average delta; Fig. 2A, middle panel). By comparison, for male participants, the modification added a relatively low FFE improvement (4.0–9.7%) over baseline (Table 3; Fig. 2A, right panel). The largest increase in modified FFE was observed with the KF94 face mask for female participants ( + 26.1%). In the mixed effects model, sex and the interaction term with sex and clip were significant predictors (*p* < 0.0001). Across all masks, males, on average, are estimated to have an FFE improvement using the clip that is −13.6% (95% CI −16.4, −10.7) lower than females based on the interaction term in the model.

As shown in Fig. 2B, the FFE improved among males and females for KN95, surgical, and KF94 face masks, with clip modification. Overall, the FFE for most females benefited from implementation of the clip modification. Although on average the clip-modified ear loop face masks improved FFE, there were several instances, mainly among males, where using the clip reduced FFE relative to baseline. Individual participant variation can be viewed in greater detail in the density cloud plots (Fig. 3B–D), which show the distribution of individuals exhibiting improvement or decline in FFE with clip-modified relative to baseline. Paired t-tests show a significant (*p* < 0.0001) difference between the improvement with the clip modification between females and males for all masks. Relatively modest improvements in the FFE of the KN95 ( + 4.0%; Fig. 3B) and surgical ( + 4.1%; Fig. 3C) masks were observed for males when modified with an ear loop clip, with a slightly larger improvement for the KF94 mask ( + 9.7%; Fig. 3D). In striking contrast, the addition of an ear loop clip resulted in robust improvements in the FFE of female participants for KN95 ( + 18.8%; Fig. 3B), surgical ( + 13.7%; Fig. 3C), and KF94 masks ( + 26.1%; Fig. 3D).

## DISCUSSION

This study highlighted the magnitude of variability in baseline fitted filtration efficiency (FFE) across participants for commonly worn ear loop disposable masks. Consistent with previous results, N95 respirator results exhibited the highest FFE compared to all other masks and the lowest variability between participants. However, in the context of the COVID-19 pandemic, N95 respirators are reported to have been worn infrequently relative to other types of face masks or respirators, with KN95 and surgical masks comprising the most commonly worn face coverings by the American public [16]. Given their availability, relative comfort, and low cost, it is likely that these ear loop masks will remain relevant during future emergencies. Therefore, it is important to characterize the factors that contribute to variation in the protection that these disposable face masks provide. We identify sex as a major determinant of the fitted protection that ear loop face masks, including KN95, surgical, and KF94 models, provide. We show that males have significantly better FFE at baseline for all disposable ear loop masks tested.

The high amount of variation in baseline FFE data for ear loop face masks (KN95, surgical, and KF94) highlights extensive inter-participant differences. Disposable respirators are available in Europe as filtering face piece (FFP) masks and in China and South Korea as KN95 and KF94, respectively. FFP categories are contingent on the filtering efficiency of particles > 0.3 um: FFP1 ( > 80%), FFP2 ( > 94%) and FFP3 ( > 99%), which are intended to provide increasing levels of protection [18–20]. However, the different face mask standards are based on mask material performance and therefore do not represent the filtering efficiency as fitted on an individual. In the U.S., N95 respirators are the only type of disposable facemasks currently subject to occupational fit testing protocols. While we show the N95 always exceeded the FFP1 threshold (80%), ear loop mask performance was less consistent, with no masks of this style having reliably exceeded this threshold. The KN95 was the next highest FFE level mask, with males consistently outperforming females. This pattern proved consistent with males also exhibiting higher baseline FFE in surgical and KF94 masks, compared to females.

Other notable between-sex patterns emerged when using a simple ear loop clip where larger FFE improvements were observed for females compared to males. Despite having lower baseline FFE for all ear loop masks compared to males, modifying the masks with a clip allowed females to equal or exceed the FFE of males. For the KN95, females had an average of 84.5% which was the only ear loop masking condition to exceed the FFP1 threshold. In some instances, the use of an ear loop clip for males was even detrimental to fit. The findings of this study suggest that when using an ear loop clip, most women improve the protection afforded by KN95, surgical, and KF94 masks. Males, however, show far less benefit using a clip, especially when applied to the KN95 where they are more likely to reduce their FFE in the modified vs. baseline condition.

While females tend to benefit greatly when using an ear loop clip, our data also suggests that gains in FFE decrease when approaching a mask’s performance threshold. When the clip-modified FFE change was evaluated by quartile of baseline performance, significant differences were found between the bottom quartile compared to quartiles two, three, and four. Given that modification has greater improvement potential for lower baseline levels, it is not surprising that there were relatively minor improvements observed in males, who started with higher overall baseline FFE values relative to female participants. We hypothesize that known sexual dimorphism in body size, and thus head size, partially contributes to the observed variation in FFE and improvement with modification seen primarily in women. In an anthropometric survey of 3,997 respirator wearers, Zhuang and Bradtmiller found higher average values in men compared to women for all cranial measurements [21]. The strong effect of sex seen in this study demonstrates that the FFE of disposable face masks is limited by complex interactions between the intrinsic dimensions of each mask and the craniofacial features of the wearer.

Limitations of this study include the fact that, for practical reasons, only a single model and manufacturer version could be tested for each mask type. Furthermore, it should be specifically noted for KN95 and KF94 masks, there is no universal sizing and that other sizes are available for these mask types. In addition, due to safety considerations related to the duration and intensity of testing, the study population was restricted in age and BMI and to healthy participants. Replication of this study with a larger and more diverse population, that expands the age and BMI restrictions of this study, and includes masks produced by different manufacturers, would corroborate, and strengthen the generalizability of these findings. Future studies are warranted to better understand and elucidate the interactions between craniofacial morphology and the FFE of commonly worn masks, which ultimately may be used to guide the selection of masks and fit enhancements on an individual level.

This study characterized the fitted performance of commonly used face masks, identifying a sex-based disparity in ear loop types that has important public health implications. Furthermore, the findings indicate that a simple modification, the use of an ear loop clip, can reverse the disparity observed. This dichotomy represents a differential benefit in the protection that can be achieved for females against infectious or toxic respiratory pollutants. Thus, modifying fit can potentially serve as a mask performance equalizer, considering the reduced baseline level observed for female participants, relative to males. Given a lack of widely available fit testing, these findings can provide guidance to the public on what masks to wear and whether they will benefit from the simple “hack” of using an ear loop clip fastened behind the head. This information can improve risk reducing behaviors and help to increase protection against toxic aerosols, an important consideration given the increasing prevalence of air quality emergencies across the globe.

## Supplementary Material

Supplement1

## Figures and Tables

**Table 1.** Participant demographics and BMI.

	Participants				F vs M
	All		Female	Male	
**Number**	100		50	50	
**Age (yrs)**					
Average (s.d.)	32.3 (9.2)		33.4 (9.2)	31.1 (9.0)	n.s.
**Height (cm)**					
Average (s.d.)	170.6 (9.7)		163.8 (6.2)	177.4 (7.5)	***
**Weight (kg)**					
Average (s.d.)	74.4 (12.5)		69.2 (11.3)	79.5 (11.6)	***
**Body Mass Index (kg/m^2^)**					
Average (s.d.)	25.5 (3.4)		25.8 (3.8)	25.2 (3.0)	n.s.
**Race/Ethnicity**	*n*	%			
Asian	19	0.19			
African American	17	0.17			
Hispanic/Latino	5	0.05			
Native American	1	0.01			
Caucasian	60	0.60			

Female (F) vs Male (M) t-test:

*** *p* < 0.001; *n.s.* not significant.

**Table 2.** Fitted filtration efficiency (FFE) of tested face masks (*n* = 100, Female = 50, Male = 50).

	Baseline Fitted Filtration Efficiency (FFE)					
		
	1st (25%)	2nd (50%)	3rd (75%)	Average/median (50%) values		
	Participants					*Tukey HSD tests*
		
	All			Female	Male	F vs M

**N95 Respirator**						

Average FFE (s.d.)		97.8 (3.2)		97.4 (3.4)	98.2 (2.9)	n.s.

Quartile	97.7	99.2	99.6	98.8	99.5	

**KN95**						

Average FFE (s.d.)		69.4 (12.2)		65.7 (12.0)	73.1 (11.5)	***

Quartile	60.5	70.2	78.6	64.2	74.7	

**Surgical**						

Average FFE (s.d.)		57.5 (9.9)		52.4 (8.0)	62.5 (9.2)	***

Quartile	49.6	57.7	63.6	52.3	62.3	

**KF94**						

Average FFE (s.d.)		55.2 (15.6)		47.6 (13.8)	62.8 (13.5)	***

Quartile	43.0	52.8	67.3	45.0	62.0	

Female (F) vs Male (M) t-test:

*** *p* < 0.001; *n.s.* not significant.

**Table 3.** Fitted filtration efficiency (FFE) of ear loop face masks with a clip secured behind the head.

	Clip-modified Fitted Filtration Efficiency (FFE)						
		
	Participants						*Tukey HSD tests*
		
	All	*delta*	Female	*delta*	Male	*delta*	F vs M

**KN95**							

Average FFE (s.d.)	80.8 (12.0)	*11.4*	84.5 (10.0)	*18.8*	77.0 (12.6)	*4.0*	***

Median	82.1	*9.9*	87.0	*19.1*	75.6	*5.2*	

**Surgical**							

Average FFE (s.d.)	66.4 (9.4)	*8.9*	66.1 (7.8)	*13.7*	66.6 (10.8)	*4.1*	***

Median	67.9	*8.8*	67.6	*14.4*	68.4	*3.9*	

**KF94**							

Average FFE (s.d.)	73.1 (13.9)	*17.9*	73.7 (13.8)	*26.1*	72.5 (14.2)	*9.7*	***

Median	75.6	*15.7*	77.0	*26.4*	71.4	*10.3*	

Delta (subtraction change versus baseline level) shown in italics.

Female (F) vs Male (M) t-test:

*** *p* < 0.001.

## Data Availability

This research was conducted by the U.S. Environmental Protection Agency and as such data will be available through ScienceHub at https://catalog.data.gov/dataset/epa-sciencehub.
