# Supplementary material for: Variation in the fitted filtration efficiency of disposable face masks by sex
Source: J Expo Sci Environ Epidemiol. 2024 Jul 2;35(4):683–90. doi: 10.1038/s41370-024-00697-4 (PMC11693769; doi:10.1038/s41370-024-00697-4)
Supplement: Supplementary file 1 — supplemental stat analysis [file 41370_2024_697_MOESM1_ESM.docx]

Face Fit Statistical Analysis

**Experiment 1:**

Goal: The primary goal of this study was to investigate the variance in FFE of respiratory masks (i.e.,N95, KN95, surgical mask, and KF94) commonly worn by the public, relative to the overall mean.

*Final Model (Significance level is alpha = 0.05 for all models)*

## Linear mixed-effects model fit by REML

## Data: FFE_base

## AIC BIC logLik

## 2946.576 2986.289 -1463.288

##

## Random effects:

## Formula: ~1 | Subject

## (Intercept) Residual

## StdDev: 5.506446 8.466227

##

## Fixed effects: Overall_FFE ~ Condition * Sex

## Value Std.Error DF t-value p-value

## (Intercept) 69.970 0.6945517 294 100.74125 0.0000

## Condition1 27.855 0.7331968 294 37.99116 0.0000

## Condition2 -0.575 0.7331968 294 -0.78424 0.4335

## Condition3 -12.514 0.7331968 294 -17.06772 0.0000

## Sex1 -4.184 0.6945517 98 -6.02403 0.0000

## Condition1:Sex1 3.763 0.7331968 294 5.13232 0.0000

## Condition2:Sex1 0.505 0.7331968 294 0.68876 0.4915

## Condition3:Sex1 -0.846 0.7331968 294 -1.15385 0.2495

## Correlation:

## (Intr) Cndtn1 Cndtn2 Cndtn3 Sex1 Cn1:S1 Cn2:S1

## Condition1 0.000

## Condition2 0.000 -0.333

## Condition3 0.000 -0.333 -0.333

## Sex1 0.000 0.000 0.000 0.000

## Condition1:Sex1 0.000 0.000 0.000 0.000 0.000

## Condition2:Sex1 0.000 0.000 0.000 0.000 0.000 -0.333

## Condition3:Sex1 0.000 0.000 0.000 0.000 0.000 -0.333 -0.333

##

## Standardized Within-Group Residuals:

## Min Q1 Med Q3 Max

## -2.35457594 -0.57955538 -0.04891315 0.58237664 3.35308253

##

## Number of Observations: 400

## Number of Groups: 100

Comparing the fixed effects and mixed effects models

Model BIC

model3 (mixed) 2986.289

model2 (fixed) 3010.467

*Confidence Intervals*

For the estimated coefficients of the mixed effects model

Approximate 95% confidence intervals

Fixed effects:

lower est. upper

(Intercept) 68.6030767 69.970 71.3369233

Condition1 26.4120205 27.855 29.2979795

Condition2 -2.0179795 -0.575 0.8679795

Condition3 -13.9569795 -12.514 -11.0710205

Sex1 -5.5623151 -4.184 -2.8056849

Condition1:Sex1 2.3200205 3.763 5.2059795

Condition2:Sex1 -0.9379795 0.505 1.9479795

Condition3:Sex1 -2.2889795 -0.846 0.5969795

Random Effects:

Level: Subject

lower est. upper

sd((Intercept)) 4.384673 5.506446 6.915214

Within-group standard error:

lower est. upper

7.808848 8.466227 9.178948

*Sensitivity Analysis*

## Linear mixed-effects model fit by REML

## Data: FFE_base

## AIC BIC logLik

## 2930.102 2993.395 -1449.051

##

## Random effects:

## Formula: ~1 | Subject

## (Intercept) Residual

## StdDev: 4.961273 8.466228

##

## Fixed effects: Overall_FFE ~ Condition * Sex + Race_Ethnicity + Age + BMI

## Value Std.Error DF t-value

## (Intercept) 54.98314 5.349393 294 10.27839

## Condition1 27.85500 0.733197 294 37.99116

## Condition2 -0.57500 0.733197 294 -0.78424

## Condition3 -12.51400 0.733197 294 -17.06772

## Sex1 -4.33923 0.709307 92 -6.11756

## Race_EthnicityHispanic/Latino -3.58601 3.322273 92 -1.07938

## Race_EthnicityNon-Hispanic Black 2.30556 2.352730 92 0.97995

## Race_EthnicityNon-Hispanic White -1.27099 1.851677 92 -0.68640

## Race_EthnicityOther including Multi-Race 7.71563 4.910332 92 1.57130

## Age 0.16897 0.073542 92 2.29757

## BMI 0.38807 0.186866 92 2.07671

## Condition1:Sex1 3.76300 0.733197 294 5.13232

## Condition2:Sex1 0.50500 0.733197 294 0.68876

## Condition3:Sex1 -0.84600 0.733197 294 -1.15385

## p-value

## (Intercept) 0.0000

## Condition1 0.0000

## Condition2 0.4335

## Condition3 0.0000

## Sex1 0.0000

## Race_EthnicityHispanic/Latino 0.2832

## Race_EthnicityNon-Hispanic Black 0.3297

## Race_EthnicityNon-Hispanic White 0.4942

## Race_EthnicityOther including Multi-Race 0.1195

## Age 0.0239

## BMI 0.0406

## Condition1:Sex1 0.0000

## Condition2:Sex1 0.4915

## Condition3:Sex1 0.2495

## Correlation:

## (Intr) Cndtn1 Cndtn2 Cndtn3 Sex1

## Condition1 0.000

## Condition2 0.000 -0.333

## Condition3 0.000 -0.333 -0.333

## Sex1 0.187 0.000 0.000 0.000

## Race_EthnicityHispanic/Latino -0.025 0.000 0.000 0.000 -0.091

## Race_EthnicityNon-Hispanic Black -0.028 0.000 0.000 0.000 -0.260

## Race_EthnicityNon-Hispanic White -0.182 0.000 0.000 0.000 -0.329

## Race_EthnicityOther including Multi-Race -0.009 0.000 0.000 0.000 0.052

## Age -0.392 0.000 0.000 0.000 -0.047

## BMI -0.864 0.000 0.000 0.000 -0.089

## Condition1:Sex1 0.000 0.000 0.000 0.000 0.000

## Condition2:Sex1 0.000 0.000 0.000 0.000 0.000

## Condition3:Sex1 0.000 0.000 0.000 0.000 0.000

## R_EH/L R_EN-B R_EN-W R_EOiM Age

## Condition1

## Condition2

## Condition3

## Sex1

## Race_EthnicityHispanic/Latino

## Race_EthnicityNon-Hispanic Black 0.333

## Race_EthnicityNon-Hispanic White 0.402 0.629

## Race_EthnicityOther including Multi-Race 0.148 0.187 0.222

## Age 0.000 -0.127 -0.131 0.090

## BMI -0.126 -0.141 -0.024 -0.124 -0.020

## Condition1:Sex1 0.000 0.000 0.000 0.000 0.000

## Condition2:Sex1 0.000 0.000 0.000 0.000 0.000

## Condition3:Sex1 0.000 0.000 0.000 0.000 0.000

## BMI Cn1:S1 Cn2:S1

## Condition1

## Condition2

## Condition3

## Sex1

## Race_EthnicityHispanic/Latino

## Race_EthnicityNon-Hispanic Black

## Race_EthnicityNon-Hispanic White

## Race_EthnicityOther including Multi-Race

## Age

## BMI

## Condition1:Sex1 0.000

## Condition2:Sex1 0.000 -0.333

## Condition3:Sex1 0.000 -0.333 -0.333

##

## Standardized Within-Group Residuals:

## Min Q1 Med Q3 Max

## -2.36653838 -0.62053102 -0.00874441 0.58111595 3.22887051

##

## Number of Observations: 400

## Number of Groups: 100

**Experiment 2:**

Goal: In addition, we aimed to determine whether a simple modification, using an ear loop clip worn behind the head, improves the FFE of these masks.

N95 removed from analysis because it cannot be modified by a clip. Only ear loop masks are included. Addition of the clip is considered a modification.

*Models (all models use a significance level of 0.05)*

All models are mixed effects model with Subject as the random effect, since mixed effects model was the best fit for Experiment 1. Clip is the new variable (mask modification); a binary variable where 1 indicates present and 0 indicates absent. The model compares the overall mean.

## Linear mixed-effects model fit by REML

## Data: FFE_clip_subset

## AIC BIC logLik

## 4504.295 4539.39 -2244.148

##

## Random effects:

## Formula: ~1 | Subject

## (Intercept) Residual

## StdDev: 7.902941 8.875643

##

## Fixed effects: Overall_FFE ~ Condition + Clip * Sex

## Value Std.Error DF t-value p-value

## (Intercept) 67.04883 0.8694020 496 77.12064 0.0000

## Condition1 -5.14233 0.5124355 496 -10.03508 0.0000

## Condition2 -2.89633 0.5124355 496 -5.65209 0.0000

## Clip1 -6.36383 0.3623466 496 -17.56283 0.0000

## Sex1 -2.04383 0.8694020 98 -2.35085 0.0207

## Clip1:Sex1 -3.39450 0.3623466 496 -9.36810 0.0000

## Correlation:

## (Intr) Cndtn1 Cndtn2 Clip1 Sex1

## Condition1 0.0

## Condition2 0.0 -0.5

## Clip1 0.0 0.0 0.0

## Sex1 0.0 0.0 0.0 0.0

## Clip1:Sex1 0.0 0.0 0.0 0.0 0.0

##

## Standardized Within-Group Residuals:

## Min Q1 Med Q3 Max

## -2.65740780 -0.67103702 0.06357832 0.66793099 2.26519016

##

## Number of Observations: 600

## Number of Groups: 100

## Approximate 95% confidence intervals

##

## Fixed effects:

## lower est. upper

## (Intercept) 65.340669 67.048833 68.7569980

## Condition1 -6.149145 -5.142333 -4.1355215

## Condition2 -3.903145 -2.896333 -1.8895215

## Clip1 -7.075757 -6.363833 -5.6519099

## Sex1 -3.769133 -2.043833 -0.3185334

## Clip1:Sex1 -4.106423 -3.394500 -2.6825765

##

## Random Effects:

## Level: Subject

## lower est. upper

## sd((Intercept)) 6.667941 7.902941 9.366681

##

## Within-group standard error:

## lower est. upper

## 8.340157 8.875643 9.445509

*Sensitivity Analysis*

Linear mixed-effects model fit by REML

Data: FFE_clip_subset

AIC BIC logLik

4485.04 4546.314 -2228.52

Random effects:

Formula: ~1 | Subject

(Intercept) Residual

StdDev: 7.31109 8.875643

Fixed effects: Overall_FFE ~ Condition + Clip * Sex + Race_Ethnicity + BMI + Age

Value Std.Error DF t-value p-value

(Intercept) 49.91310 6.692924 496 7.457593 0.0000

Condition1 -5.14233 0.512435 496 -10.035085 0.0000

Condition2 -2.89633 0.512435 496 -5.652094 0.0000

Clip1 -6.36383 0.362347 496 -17.562835 0.0000

Sex1 -2.12881 0.887454 92 -2.398781 0.0185

Race_EthnicityHispanic/Latino -5.60036 4.156681 92 -1.347316 0.1812

Race_EthnicityNon-Hispanic Black 2.22512 2.943632 92 0.755910 0.4516

Race_EthnicityNon-Hispanic White -2.39751 2.316737 92 -1.034864 0.3034

Race_EthnicityOther including Multi-Race 9.19231 6.143591 92 1.496244 0.1380

BMI 0.43396 0.233798 92 1.856138 0.0666

Age 0.22203 0.092012 92 2.413006 0.0178

Clip1:Sex1 -3.39450 0.362347 496 -9.368102 0.0000

Correlation:

(Intr) Cndtn1 Cndtn2 Clip1 Sex1 R_EH/L R_EN-B R_EN-W R_EOiM BMI Age

Condition1 0.000

Condition2 0.000 -0.500

Clip1 0.000 0.000 0.000

Sex1 0.187 0.000 0.000 0.000

Race_EthnicityHispanic/Latino -0.025 0.000 0.000 0.000 -0.091

Race_EthnicityNon-Hispanic Black -0.028 0.000 0.000 0.000 -0.260 0.333

Race_EthnicityNon-Hispanic White -0.182 0.000 0.000 0.000 -0.329 0.402 0.629

Race_EthnicityOther including Multi-Race -0.009 0.000 0.000 0.000 0.052 0.148 0.187 0.222

BMI -0.864 0.000 0.000 0.000 -0.089 -0.126 -0.141 -0.024 -0.124

Age -0.392 0.000 0.000 0.000 -0.047 0.000 -0.127 -0.131 0.090 -0.020

Clip1:Sex1 0.000 0.000 0.000 0.000 0.000 0.000 0.000 0.000 0.000 0.000 0.000

Standardized Within-Group Residuals:

Min Q1 Med Q3 Max

-2.65173830 -0.66347518 0.06047191 0.69189103 2.16512469

Number of Observations: 600

Number of Groups: 100

## # Groups: Condition [3]

## Condition F M estimate CI_lower CI_upper t_statistic p.value

## <chr> <dbl> <dbl> <dbl> <dbl> <dbl> <dbl> <dbl>

## 1 KF94 26.1 9.73 16.3 12.2 20.5 7.87 2.99e-10

## 2 KN95 18.8 3.98 14.8 11.3 18.3 8.59 2.43e-11

## 3 Surgical 13.7 4.11 9.59 7.56 11.6 9.49 1.10e-12

*Improvement analysis with quantiles*

## Linear mixed-effects model fit by REML

## Data: FFE_quant_long

## AIC BIC logLik

## 2217.897 2243.706 -1101.948

##

## Random effects:

## Formula: ~1 | Subject

## (Intercept) Residual

## StdDev: 5.773388 8.357928

##

## Fixed effects: diff ~ FFE + quantile

## Value Std.Error DF t-value p-value

## (Intercept) 31.581207 3.195600 196 9.882716 0.0000

## FFE -0.241812 0.064567 196 -3.745128 0.0002

## quantile2 -3.379661 1.705619 196 -1.981486 0.0489

## quantile3 -6.403687 2.025779 196 -3.161098 0.0018

## quantile4 -6.882007 2.634816 196 -2.611950 0.0097

## Correlation:

## (Intr) FFE quntl2 quntl3

## FFE -0.918

## quantile2 0.123 -0.394

## quantile3 0.358 -0.618 0.631

## quantile4 0.519 -0.757 0.604 0.758

##

## Standardized Within-Group Residuals:

## Min Q1 Med Q3 Max

## -2.31682788 -0.62119389 -0.01485066 0.56234614 2.67128268

##

## Number of Observations: 300

## Number of Groups: 100

## numDF denDF F-value p-value

## (Intercept) 1 196 286.12167 <.0001

## FFE 1 196 83.52543 <.0001

## quantile 3 196 3.37125 0.0196

## Approximate 95% confidence intervals

##

## Fixed effects:

## lower est. upper

## (Intercept) 25.2790324 31.5812071 37.88338182

## FFE -0.3691469 -0.2418117 -0.11447643

## quantile2 -6.7433828 -3.3796607 -0.01593872

## quantile3 -10.3988103 -6.4036871 -2.40856387

## quantile4 -12.0782362 -6.8820070 -1.68577776

##

## Random Effects:

## Level: Subject

## lower est. upper

## sd((Intercept)) 4.502805 5.773388 7.402499

##

## Within-group standard error:

## lower est. upper

## 7.570475 8.357928 9.227289
